# Outcomes of mechanical ventilation according to WIND classification in pediatric patients

**DOI:** 10.1186/s13613-019-0547-2

**Published:** 2019-06-27

**Authors:** Ah Young Choi, Minji Kim, Esther Park, Meong Hi Son, Jeong-Am Ryu, Joongbum Cho

**Affiliations:** 1 Department of Critical Care Medicine, Samsung Medical Center, Sungkyunkwan University School of Medicine, 81 Irwon-ro, Gangnam-gu, Seoul, 06351 Republic of Korea; 2 Department of Pediatrics, Hallym University Dongtan Sacred Heart Hospital, Hallym University College of Medicine, Hwaseong, Republic of Korea; 3 Department of Pediatrics, Samsung Medical Center, Sungkyunkwan University School of Medicine, 81 Irwon-ro, Gangnam-gu, Seoul, 06351 Republic of Korea

**Keywords:** Mechanical ventilation, Ventilator weaning, Pediatric intensive care unit, Prognosis, Epidemiology, Classification

## Abstract

**Background:**

The outcomes of weaning processes are not well known in pediatric patients, and the International Conference Classification on weaning from mechanical ventilation showed limited application. We evaluate the relationship between the new Weaning according to a New Definition (WIND) classification and outcome in pediatric patients.

**Methods:**

We conducted a retrospective cohort study in a tertiary pediatric intensive care unit (ICU). We included patients under 18 years of age who received invasive mechanical ventilation for more than 24 h and excluded cases with other than the first ICU admissions, tracheostomy with home ventilation before admission, intubation or weaning processes conducted in other ICU, and weaning with extracorporeal membrane oxygenation. Weaning processes were classified into four groups according to weaning duration after the first separation attempt (SA): no-SA, short weaning (< 24 h), difficult weaning (24 h–7 days), and prolonged weaning (> 7 days). Mortality rates were compared across groups using the Kruskal–Wallis test, and risk factors for the no-SA group were analyzed by multivariate logistic regression tests with age, sex, severity score at admission, admission type, and underlying disease as variables.

**Results:**

Among 313 patients, 224 were enrolled and had a median age of 2.1 (interquartile range 0.5–6.6) years. Spontaneous breathing tests were done in 70.1% of enrolled patients. The median duration of intubation to the first SA was 4 (range 0–36) days, and 92.8% patients underwent the first SA within 14 days. The mortality rate was 0% in the short (0/99) and difficult (0/53) weaning groups and 17.9% (5/28) in the prolonged weaning group (*p *< 0.001). The mortality rate of the no-SA group was 93.2% (41/44). Admission severity (hazard ratio 1.036, confidence interval 1.022–1.050) and underlying oncologic disease (hazard ratio 7.341, confidence interval 3.008–17.916) were independent risk factors for lack of SA.

**Conclusions:**

In conclusion, WIND classification is associated with ICU mortality in pediatric patients. Further studies of this association are required to improve protocols associated with the weaning process and clinical outcomes.

*Trial registration* Retrospectively registered.

**Electronic supplementary material:**

The online version of this article (10.1186/s13613-019-0547-2) contains supplementary material, which is available to authorized users.

## Background

Mechanical ventilation (MV) is a widely used form of respiratory support in pediatric intensive care units (ICUs). However, prolonged use of MV causes complications [1–3]. The weaning period may comprise up to 40% of MV days [4, 5]. Weaning from MV is difficult for 30% of patients, and such patients showed a higher mortality rate [6]. Therefore, previous studies have assessed the relationships between weaning classifications and clinical outcomes.

The weaning process was classified by the International Consensus Conference on weaning from MV according to difficulty and duration of the weaning process: simple weaning, difficult weaning, and prolonged weaning [7]. Patients with prolonged weaning have higher mortality than other groups [8–11]. However, the International Consensus Conference classification excludes patients who were not weaned from MV or who were weaned from MV without a spontaneous breathing test (SBT). In 2017, a new pragmatic classification of weaning, the Weaning according to a New Definition (WIND) classification, was proposed to overcome these limitations and was associated with survival to discharge in adult ICU patients [12].

Studies of the weaning processes and patient outcomes in pediatric ICU patients are rare. There are some data indicating an association between extubation failure and mortality rate [13], but weaning is more complex than single extubation and requires more time. In this study, we evaluate the relationship between weaning process as categorized by WIND classification and ICU mortality in pediatric ICU patients.

## Methods

### Study design and patients

We conducted a retrospective cohort study of patients who were admitted to the pediatric intensive care unit of a single tertiary hospital in South Korea from January 2015 to October 2017. This unit consists of 15 beds and is a combined medical and surgical unit. Immediate post-cardiac surgery patients are admitted to a separate dedicated cardiac surgical unit. In the studying pediatric ICU, daily screening for readiness to wean was done for patients with mechanical ventilation for more than 24 h. Screening criteria were (1) hemodynamic stability, (2) adequate mentation with spontaneous inspiratory effort, (3) adequate oxygenation and ventilation (oxygen saturation > 90% on fraction of inspired oxygen ≤ 0.4 and pH > 7.30), and (4) not worsening chest x-ray. Spontaneous breathing test (SBT) was determined on a daily round, and T-piece, continuous positive airway pressure (4–5 cm H_2_O), or pressure support (6–8 cm H_2_O, mandatory for patients under 12 months of age) was applied for 30–120 min according to physicians’ decision. Ventilator mode or setting before weaning was not protocolized except low tidal volume for acute respiratory distress syndrome.

We screened all consecutive admissions to the pediatric ICU during the study period. We included patients under the age of 18 years who had undergone invasive mechanical ventilation for more than 24 h in the pediatric ICU. For patients with multiple ICU admissions, we included only the first ICU admission during a single hospitalization and excluded patients who had tracheostomy with home ventilation prior to ICU admission, who were intubated before transfer from other ICU or hospital, who were transferred to another hospital or ICU before they were ready to wean, and who were weaned from invasive mechanical ventilation with extracorporeal membrane oxygenation.

### Definition of the weaning process

We used the definitions and classification of the weaning process according to the WIND study [12]. For intubated patients, we defined separation attempt (SA) as an SBT with or without extubation or direct extubation without SBT. Successful weaning or separation was defined as extubation without death or reintubation regardless of noninvasive mechanical ventilation or ICU discharge within the next 7 days. The SBT included breathing with (1) a T-piece trial, (2) pressure support ≤ 8 cm H_2_O, or (3) continuous positive airway pressure ≤ 5 cm H_2_O. For tracheostomized patients, SA was defined as spontaneous ventilation through tracheostomy without any mechanical ventilation for more than 24 h. Successful weaning for tracheostomized patients was defined as spontaneous ventilation through tracheostomy without any mechanical ventilation for 7 days or discharged with spontaneous breathing.

We classified patients into four groups according to duration of the weaning process following the WIND study. The no-separation attempt group (group no-SA) was comprised of patients who never underwent SA (any SBT or extubation trial). The short weaning group (group 1) had weaning processes that were terminated within 24 h after the first SA. The difficult weaning group (group 2) had weaning processes terminated after more than 24 h but in less than 7 days after the first SA. The prolonged weaning group (group 3) had weaning processes not terminated 7 days after the first SA.

### Data collection and statistical analysis

Data for patients’ clinical history, changes in ventilator settings, and event records including SBT, extubation, and intubation trials were reviewed and collected by intensive care fellows from electronic medical records. Baseline patient characteristics of age, sex, underlying disease, reasons for ICU admission, and illness severity were measured, the latter of which was measured by the pediatric index of mortality 3 (PIM 3) score. We measured the weaning process parameters of time from intubation to the first SA, time from the first SA to successful weaning, total length of invasive mechanical ventilation, implementation of SBT/technique of SBT, and use of respiratory support after extubation. We measured clinical outcomes of ICU mortality, weaning status at ICU discharge, length of ICU stay, length of hospital stay, and ventilator-free days (the number of days survived without mechanical ventilation to day 28).

Descriptive data are presented as frequencies (percentages) for categorical variables and medians and interquartile ranges for continuous variables. The normality of continuous variables was tested by Shapiro–Wilks test. Proportions of sex, admission type, reason for ICU admission, underlying disease, reintubation, tracheostomy rate, and ICU mortality were compared using Chi-squared or Fisher’s exact tests. Continuous variables of age, PIM 3 score, duration of invasive MV, ventilator-free days, duration from intubation to the first SA, length of stay in the ICU, length of stay in the hospital, and ICU-free days (the number of days survived without ICU admission to day 28) were analyzed using the Mann–Whitney *U* test for comparisons of two groups (SA vs. no-SA) and were analyzed using the Kruskal–Wallis test for comparisons of three groups (among groups 1, 2, and 3, not included no-SA group). Multivariable logistic regression was performed to analyze factors associated with lack of SA. Variables included in the regression test were age, sex, PIM 3 score at admission, admission type (surgical or medical), and presence of underlying oncologic disease, and they were included for clinical relevance or selected when *p* value was below 0.2 in univariate analysis. Statistical analyses were performed with SPSS version 22.0 software (IBM Inc., Armonk, NY, USA).

## Results

**Fig. 1:**
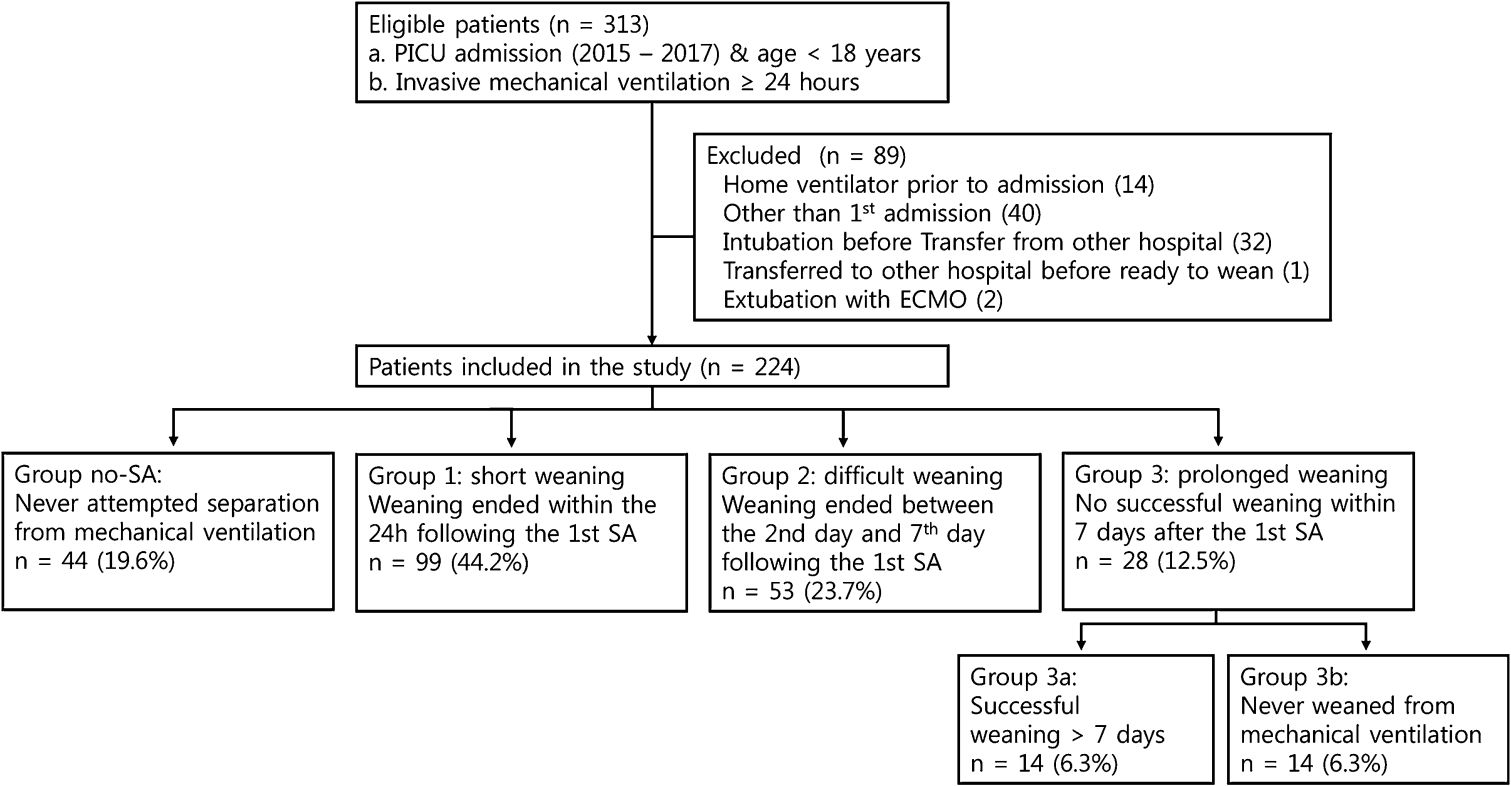
Flow diagram of patient selection, with inclusion and exclusion criteria and weaning classifications. *PICU* pediatric intensive care unit, *ECMO* extracorporeal membrane oxygenation, *SA* separation attempt

During the study period, 313 patients were admitted to the pediatric ICU and required MV for more than 24 h. Among these patients, 89 were excluded according to exclusion criteria: 14 patients had been on home ventilators at admission, 40 admissions were other than the first ICU admission, 32 patients were transferred from other hospitals with intubation, two patients were extubated on extracorporeal membrane oxygenation, and one patient was transferred to another hospital before ready to wean. Therefore, 224 patients were included in the final analysis (Fig. 1).

For baseline characteristics, the median age of the 224 enrolled patients was 2.1 (interquartile range 0.5–6.6) years (Table 1). The median-predicted mortality rate at admission was 4.6% (interquartile range [IQR] 1.4–15.0) with right-skewed distribution. The mean predicted mortality rate was 15.5%, and the actual mortality rate was 20.5% (46/224). The standardized mortality ratio calculated by observed/predicted (PIM 3) mortality was 1.32 (95% confidence interval [CI] 0.75–1.89). Thirty-seven (16.5%) patients were admitted for postoperative care, and 187 (83.5%) patients were admitted for medical organ dysfunctions or problems. The most common underlying condition was oncologic disease (21.9%), followed by cardiologic (21.0%) and neurologic disease (21.0%) (Table 1).

**Table 1:** Patient characteristics and outcomes according to separation attempt

	Total patients (*n* = 224)	SA group (*n* = 180)	No-SA group (*n* = 44)	*p* value
*Baseline characteristics*				
Age in years	2.1 (0.5–6.6)	1.6 (0.4–5.5)	5.3 (1.2–13.0)	0.001
Male	122 (54.5)	102 (56.7)	20 (45.5)	0.121
PIM 3 (%), median (IQR)	4.6 (1.4–15.0)	3.0 (0.9–8.2)	22.3 (7.0–84.0)	<0.001
PIM 3 (%), mean (SD)	15.5 (26.0)	10.5 (20.0)	36.2 (36.0)	
Admission type				
Postoperative admission	37 (16.5)	35 (19.4)	2 (4.5)	0.010
Medical admission	187 (83.5)	145 (80.6)	42 (95.5)	0.009
Respiratory	117 (52.2)	98 (67.6)	19 (45.2)	
Cardiovascular	33 (14.7)	21 (14.5)	12 (28.6)	
Neurologic	20 (8.9)	17 (11.7)	3 (7.1)	
Other	17 (7.7)	9 (6.2)	8 (19.1)	
Underlying disease				0.001
Neurologic	49 (21.9)	40 (22.2)	7 (15.9)	
Pulmonary	47 (21.0)	20 (11.1)	1 (2.3)	
Cardiologic	47 (21.0)	40 (22.2)	7 (15.9)	
Oncologic	21 (9.4)	27 (15.0)	22 (50.0)	
Infectious	19 (8.5)	16 (8.9)	3 (6.8)	
Other	41 (18.3)	37 (21.0)	4 (9.1)	
*Clinical outcomes*				
Total MV duration, days	7.0 (4–14)	7.0 (4–13)	12.5 (6.0–31.5)	< 0.001
Ventilator-free days^a^	19 (0–24)	21 (15–24)	0	< 0.001
Length of ICU stay, days	12 (7–21.7)	12.0 (7–21)	13.0 (6.0–31.7)	0.448
ICU-free days^a^	13 (0–20)	16 (7–21)	0 (0–0)	< 0.001
Length of hospital stay, days	26 (14–48)	24.0 (14–48)	33.5 (13.3–58.0)	0.753
Status at ICU discharge				
Dead	46 (20.5)	5 (2.8)	41 (93.2)	< 0.001
Alive and weaned				
Spontaneous breathing	144 (64.3)	144 (80.0)	0	
Tracheostomy, no MV	22 (9.8)	22 (12.2)	0	
Alive and not weaned				
Tracheostomy with MV	12 (5.4)	9 (5.0)	3 (6.8)	

Data are presented as *n* (%) or median (interquartile range)

*SA* separation attempt, *IQR* interquartile range, *SD* standard deviation, *PIM 3* pediatric index of mortality 3, *MV* mechanical ventilation, *ICU* intensive care unit

^a^Ventilator-free days/ICU-free days were calculated as the number of days without mechanical ventilation/ICU admission to day 28. Non-survivors were calculated as a value of 0

For the characteristics of weaning, SBT was applied in 157 patients (70.1% of 224 enrolled patients and 87.2% of 180 patients with SA), and post-extubation support was applied in 36 (16.1%) patients. The median duration of mechanical ventilation was 7 (IQR 4–14) days, and ventilator-free days were 19 (IQR 0–24) days. The median duration after intubation to the first SA was 4 (range 0–36) days. The first SA was performed within 7 days after intubation in 76.7% of patients (138/180), within 14 days in 92.8% of patients (167/180), and within 28 days in 99.4% (179/180) of patients. The median duration after the first SA to successful weaning was 0.83 (IQR 0.1–2.14) days. Weaning was done with tracheostomy in 22 patients (9.8%), although 12 patients (5.4%) with tracheostomy could not wean mechanical ventilation.

In the WIND classification, 99 (44.2%) completed the weaning process within 24 h and were classified as group 1. All patients in group 1 were successfully weaned. Fifty-three patients (23.7%) completed the weaning process from 1 to 7 days (group 2), and all were successfully weaned. Twenty-eight patients (12.5%) had weaning processes lasting more than 7 days (group 3). Thirteen patients in group 3 were weaned, but 15 were not. Finally, 44 (19.6%) patients who had never undergone SA were classified in the no-SA group (Fig. 1).

We compared the characteristics and clinical outcomes of the weaning groups (Table 2). The mortality rate predicted by PIM 3 score at ICU admission tended to increase from group 1 to group 3, but the difference was not significant. Age and underlying oncologic disease also tended to increase from group 1 to group 3, but the difference was not significant. As the duration of the weaning process increased in groups 1, 2, and 3, the total duration of MV, reintubation rate, time to reintubation after the first extubation, tracheostomy rate, rate of post-extubation support application, and length of stay in ICU were significantly different (Table 2). However, the duration from intubation to the first SA was shorter in group 3 than in groups 1 and 2, and application of SBT was not significantly different across groups (*p* = 0.175). Reintubation rate after the first extubation trial was high in group 3 (92.8%). Poor lung parenchymal was the most common cause (50.0%) followed by airway problem (15.4%) and problem of breathing control (15.4%), and statistical difference among groups 1, 2, and 3 was not found (*p* value = 0.106) (Additional file 1: Supplementary Table 1). ICU mortality occurred only in group 3 patients with SA (*p* value < 0.001), but group 3 was not associated with mortality in adjusted analysis conducted in SA group with variables of age, sex, PIM 3 score (*p* value = 0.996).

**Table 2:** Clinical characteristics and outcomes of patients according to weaning group

	Group 1 < 1 day (*n* = 99)	Group 2 1–7 days (*n* = 53)	Group 3 > 7 days (*n* = 28)	*p* value
*Baseline characteristics*				
Age in years	1.4 (0.4–5.3)	1.7 (0.3–4.0)	3.7 (0.9–10.8)	0.184
Male	63 (63.6)	23 (43.4)	16 (57.1)	0.057
PIM 3 (%), median (IQR)	2.4 (0.8–9.8)	3.2 (0.9–7.7)	4.6 (1.4–8.0)	0.795
Admission type				
Postoperative admission	24 (24.2)	5 (9.4)	6 (21.4)	0.087
Medical admission	75 (75.8)	48 (90.6)	22 (78.6)	
Respiratory	47 (62.7)	34 (70.8)	17 (77.3)	
Cardiovascular	14 (18.7)	6 (12.5)	1 (4.5)	
Neurologic	10 (13.3)	5 (10.4)	2 (9.1)	
Other	4 (5.3)	3 (6.3)	2 (9.1)	
Underlying disease				0.266
Neurologic	23 (23.2)	13 (24.5)	4 (14.3)	
Pulmonary	12 (12.1)	5 (9.4)	3 (10.7)	
Cardiologic	21 (21.2)	15 (28.3)	4 (14.3)	
Oncologic	8 (8.1)	11 (20.8)	8 (28.6)	
Infectious	9 (9.1)	5 (9.4)	2 (7.1)	
Other	26 (26.3)	4 (7.6)	7 (25)	
*Weaning characteristics*				
Spontaneous breathing test	83 (83.8)	50 (94.3)	24 (85.7)	0.175
PSV	78 (93.9)	45 (90.0)	20 (83.4)	
CPAP	4 (4.8)	4 (8.0)	2 (8.3)	
T-piece	1 (1.3)	1 (2.0)	2 (8.3)	
Post-extubation support	13 (13.1)	13 (24.5)	10 (35.7)	0.019
Nasal CPAP	2 (2.0)	4 (7.5)	8 (28.6)	
HFNC	11 (11.1)	9 (17.0)	2 (7.1)	
Intubation to the first SA, day	4 (2–7)	5 (3–9)	3 (2–7)	0.048
SA to successful weaning, days	0.3 (0–1)	2.6 (2–4)	17.2 (7–29)	< 0.001
Total duration of MV, days	4 (3–7)	8 (5–14)	19.5 (13–38)	< 0.001
Ventilator-free days^a^	24 (21–25)	20 (15–23)	0 (0–12)	< 0.001
Reintubation	3 (3.0)	17 (32.0)	26 (92.8)	< 0.001
Time to reintubation, h	0.2 (0.1–1.8)	1.0 (0.6–6.4)	5.7 (0.9–37.9)	0.048
*Clinical outcomes*				
Length of ICU stay, days	9 (5–14)	15 (8–23)	33.5 (18–46)	< 0.001
ICU-free day^a^	18.9 (14–23)	13.0 (5–20)	1.1 (0–10)	< 0.001
Length of hospital stay, days	18 (12–35)	31 (18–47)	49 (34–63)	0.010
Status at ICU discharge				
Dead	0	0	5 (17.9)	< 0.001
Alive and weaned	99 (100)	53 (100)	14 (50.0)	
Spontaneous breathing	92 (92.9)	43 (81.1)	9 (32.1)	
Tracheostomy, no MV	7 (7.1)	10 (18.9)	5 (17.9)	
Alive and not weaned				
Tracheostomy with MV	0	0	9 (32.1)	

Data are presented as *n* (%) or median (interquartile range)

Group no-SA had never undergone separation attempt. Group 1 underwent the weaning process that ended within the 24 h following the first SA. Group 2 underwent a weaning process that ended between the second day and the seventh day following the first SA. Group 3 underwent a weaning process 7 days or more after the first SA

*SA* separation attempt, *IQR* interquartile range*, PIM 3* pediatric index of mortality 3, *MV* mechanical ventilation, *PSV* pressure support ventilation, *CPAP* continuous positive airway pressure, *HFNC* high flow nasal cannula*, ICU* intensive care unit

^a^Ventilator-free days/ICU-free days were calculated as the number of days without mechanical ventilation/ICU admission to day 28. Non-survivors were calculated as a value of 0

In comparison of the SA groups and the no-SA group, the no-SA group was older (*p* = 0.001). The no-SA group had a higher predicted mortality rate at admission (22.3% vs. 3%, *p* < 0.001), more medical admissions (95.5% vs. 80.6%, *p* = 0.009), and a higher incidence of underlying oncologic disease (50.0% vs. 15.0%, *p* = 0/001) than the SA group. The clinical outcomes of the no-SA group included fewer ICU-free days (0 vs. 16 days, *p* < 0.001) and higher ICU mortality (93.2% vs. 2.8%, *p* < 0.001) than the SA group (Table 1). The causes of mortality were sepsis/multiorgan failure (34.8%), central nervous system failure (29.3%), respiratory failure (22.4%), and cardiac failure (7.3%) in no-SA group, and there was statistically difference between SA and no-SA groups (*p* value = 0.032) (Additional file 2: Supplementary Table 2). In a multivariate logistic regression test, the presence of underlying oncologic disease was 7.341 times higher (95% CI 3.008–17.916) in the no-SA group, and a 1% increase in PIM 3 score at admission was 1.036 times more frequent (95% CI 1.022–1.055) in the no-SA group (Table 3).

**Table 3:** Multivariate logistic regression analysis of risk factors predicting no-separation attempt

	Adjusted HR (95% CI)	*p* value
Male	1.036 (1.022–1.050)	0.434
Age	1.062 (0.987–1.142)	0.107
Medical admission	2.211 (0.454–10.762)	0.326
Oncologic disease	7.341 (3.008–17.916)	< 0.001
PIM 3 score (%)	1.036 (1.022–1.050)	< 0.001

Variables included in the regression test were age, sex, PIM 3 score at admission, admission type (surgical or medical), and presence of underlying oncologic disease

*HR* hazard ratio, *CI* confidence interval, *PIM 3* pediatric index of mortality 3

## Discussion

In this study, WIND classification according to weaning process was associated with mortality rate in pediatric patients (Additional file 3: Supplementary Fig 1). Weaning processes lasting more than 7 days were associated with increased mortality rate. About 20% of patients had never undergone SA during MV, and most deaths (89%) occurred among those patients. We demonstrated that high admission severity score and underlying oncologic disease were independent risk factors for lack of SA.

The clinical significance of the presence of SA during invasive MV was not well understood. Previous studies of weaning classifications or extubation failure enrolled only patients who underwent weaning process [3, 9, 10, 14, 15]. However, in this study, 89% mortality among mechanically ventilated patients occurred in patients who had never undergone SA. The high mortality rate in the absence of SA was similar to the results of a previous study of adults [12]. In adults, the mortality rate of the no-SA group was 86%, and the mortality of the no-SA group comprised 73.4% of total mortality. Therefore, the description of mortality rates according to WIND classification represents a comprehensive view of mortality and has pragmatic importance.

Considering the high mortality rates of no-SA group, the risk factors associated with lack of SA are clinically important. There are various causes of applying mechanical ventilation, such as acute respiratory problem, heart failure, sepsis, coma, neuromuscular problems [16]. Also, there are many reasons for weaning failures, such as airway/lung problems, brain dysfunction, cardiac dysfunction, diaphragmatic weakness, and endocrine problems [17]. There are predictive markers associated with each weaning problem in the respiratory ICU, such as weaning-induced pulmonary edema in cardiac dysfunction, thickening fraction of the diaphragm in diaphragmatic weakness, and hypercapnia [11, 18, 19]. However, these physiologic markers are not applicable to other weaning problems. In this study, severity at admission as assessed by PIM 3 score and underlying oncologic disease were independent risk factors for lack of SA. When we consider our causes of deaths and weaning criteria, our hypothesis is that the absence of SA is an indicator of uncontrolled underlying problems such as hemodynamic instability, altered mentation, or progressive respiratory failure which may inhibit weaning process and also cause death directly or indirectly. Patients with underlying oncologic disease or high PIM 3 score might be more likely to have uncontrolled underlying problems [20, 21].

Among patients who underwent SA, only group 3 (no successful weaning within 7 days) showed a significantly different mortality rate in an unadjusted analysis. According to the International Consensus Conference classification (only applying to 70% of our study population), the adjusted mortality rate in previous studies was higher in the prolonged weaning group (weaning duration longer than 7 days) than in other weaning groups, but the mortality rate was not different between simple weaning (weaning on the first attempt) and difficult weaning (weaning within 7 days) [9, 11]. Our results were similar, although we did not detect a significant difference in mortality rate in a severity-adjusted analysis.

Prolonged weaning was associated with prolonged total duration of MV in our study. In a previous study, complications such as ventilator-associated pneumonia are known to increase over time [22]. However, we did not evaluate the incidence of complications. Further studies are required to understand the association between prolonged weaning and cause of mortality.

Prolonged mechanical ventilation before weaning trial was associated with prolonged weaning in previous studies [10, 14, 23] and was also reported as a risk factor for extubation failure [15]. The results were different in our study, in which group 3 was not associated with the delayed first SA (Table 2). In the previous adult studies, T-piece or continuous positive airway pressure was common methods used for SBT, and the incidence of SBT showed a decreasing trend as weaning became more difficult [10, 14]. However, in our study, pressure support (< 8 cm H_2_O) was a common method used for SBT, and the incidence of SBT did not show any trends of increase or decrease. The different results might be caused by different study populations or different methods of SBT.

In this retrospective study design, we did not define the cutoff duration after intubation to the first SA. The duration of mechanical ventilation (range 0—36 days) before weaning trials showed a right-skewed distribution, and 99.4% (except 1 out of 180) patients tried their first SA within 28 days after intubation. Therefore, clinicians may need to consider that a patient is likely to be in the no-SA group in the absence of improvement to tolerate SA until 28 days after intubation.

We included only patients who received mechanical ventilation for more than 24 h, although there was no mention of duration in the previous WIND classification [12]. In the studying pediatric ICU, some patients applied mechanical ventilation only for several hours during a short procedure or postoperative periods. We tried to exclude them because our weaning protocol did not concern them before 24 h of mechanical ventilation. The different inclusion criteria might change the number of short weaning group (group 1).

There are several limitations to this study. First, this study was a retrospective cohort study and did not include data such as ventilator pressure settings or physiologic parameters. Second, we did not collect data about use of sedatives or neuromuscular blockers or about incidence of delirium, factors that may affect the weaning process. Third, this study was conducted in a single tertiary hospital with daily weaning assessments, and it may not apply to hospitals with low severity or different weaning characteristics. Fourth, the results of analysis using predicted mortality might be different in ICUs with a deviated standardized mortality ratio or ICUs using different severity tools. Therefore, the results may need cautions in the interpretation.

Despite these limitations, this was the first study to elucidate the outcomes of MV in pediatric patients using WIND classification. We included patients without SA or SBT, who were excluded from the previous weaning studies. Furthermore, we demonstrated the importance of the excluded population and the presence of SA as a prognostic indicator of mechanical ventilation. In addition to prognosis, development of weaning classification which describes weaning process better is clinically important, since the weaning process is also associated with ICU occupancy and healthcare cost [24]. Weaning studies based on the WIND classification might help to understand weaning process better and facilitate further research, trying to identify which population to target and which interventions to test to improve weaning protocols or policies.

In conclusion, the WIND classification of weaning from MV is associated with ICU mortality in pediatric patients. Further studies of this association are required to improve protocols associated with the weaning process and clinical outcomes.

## Additional file

**Additional file 1** Cause of reintubation after the first extubation. Poor lung parenchyma was the most common cause of reintubation, except group 1.

**Additional file 2** Sepsis/MOF was the most common cause of death. Distributionof causes according to the WIND classification was different between SA and no-SA group.

**Additional file 3** Flow diagram of WIND classification and patients' outcomes.

## Data Availability

The datasets generated during and/or analyzed during the current study are available from the corresponding author on reasonable request.

## Abbreviations

ICU: intensive care unit
MV: mechanical ventilation
PIM: pediatric index of mortality
SA: separation attempt
SBT: spontaneous breathing test
WIND: Weaning according to a New Definition
