# Supplementary material for: Outcomes of mechanical ventilation according to WIND classification in pediatric patients
Source: Ann Intensive Care. 2019 Jun 27;9:72. doi: 10.1186/s13613-019-0547-2 (PMC6597660; doi:10.1186/s13613-019-0547-2)
Supplement: Supplementary file 1 — Additional file 1 Cause of reintubation after the first extubation. Poor lung parenchyma was the most common cause of reintubation, except group 1. [file 13613_2019_547_MOESM1_ESM.docx]

Supplementary table 1. Cause of reintubation after the first extubation trial.

|  | Airway way | Lung parenchyma | Control of breathing | Hemodynamic instability | Muscle weakness | Unexpected extubation | Others |
| --- | --- | --- | --- | --- | --- | --- | --- |
| Total | 11 (23.9) | 21 (45.7) | 4 (8.7) | 1 (2.2) | 1 (2.2) | 5 (10.9) | 3 (6.5) |
| Group 1 | 1 (33.3) | 0 (0) | 0 (0) | 0 (0) | 0 (0) | 2 (66.7) | 0 (0) |
| Group 2 | 6 (35.3) | 8 (47.1) | 0 (0) | 1 (5.9) | 0 (0) | 1 (5.9) | 1 (5.9) |
| Group 3 | 4 (15.4) | 13 (50.0) | 4 (15.4) | 0 (0) | 1 (3.8) | 2 (7.7) | 2 (7.7) |
