# Supplementary material for: Outcomes of mechanical ventilation according to WIND classification in pediatric patients
Source: Ann Intensive Care. 2019 Jun 27;9:72. doi: 10.1186/s13613-019-0547-2 (PMC6597660; doi:10.1186/s13613-019-0547-2)
Supplement: Supplementary file 2 — Additional file 2 Sepsis/MOF was the most common cause of death. Distributionof causes according to the WIND classification was different between SA and no-SA group. [file 13613_2019_547_MOESM2_ESM.docx]

Supplementary table 2. Cause of deaths according to the WIND classification.

|  | Sepsis/MOF | CNS failure | Respiratory failure | Cardiac failure | Hemorrhagic shock | Total |
| --- | --- | --- | --- | --- | --- | --- |
| Total | 18 (39.1) | 12 (26.1) | 11 (23.9) | 4 (8.7) | 1 (2.2) | 46 |
| Group 1 | 0 | 0 | 0 | 0 | 0 | 0 |
| Group 2 | 0 | 0 | 0 | 0 | 0 | 0 |
| Group 3 | 2 (40.0) | 0 | 1 (20.0) | 1 (20.0) | 1 (20.0) | 5 |
| No-SA | 16 (34.8) | 12 (29.3) | 10 (24.4) | 3 (7.3) | 0 (0) | 41 |

MOF multiorgan failure, CNS central nervous system, SA separation attempt
