# Supplementary figures and images for: Outcomes of mechanical ventilation according to WIND classification in pediatric patients
Source: Ann Intensive Care. 2019 Jun 27;9:72. doi: 10.1186/s13613-019-0547-2 (PMC6597660; doi:10.1186/s13613-019-0547-2)

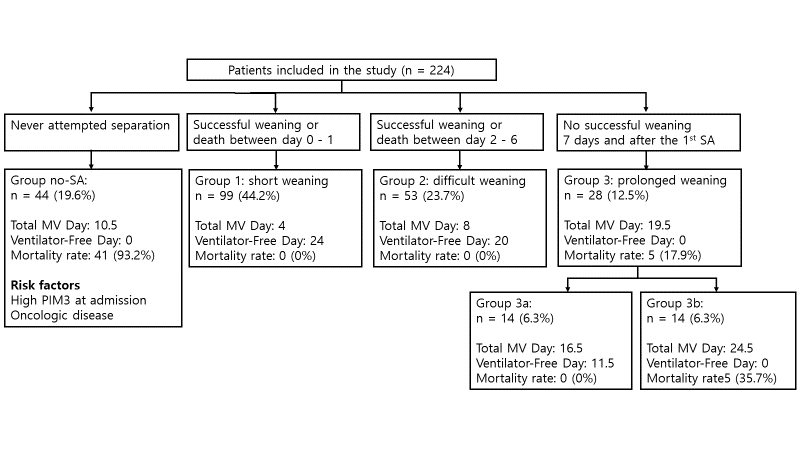
Supplementary Fig. 1. Flow diagram of WIND classification and patients’ outcomes.

Supplement: Supplementary file 3 — Additional file 3 Flow diagram of WIND classification and patients' outcomes. [file 13613_2019_547_MOESM3_ESM.docx]
